# Neuronal synchronization and multiscale information processing

**DOI:** 10.1186/1471-2202-13-S1-O14

**Published:** 2012-07-16

**Authors:** Meyer Pesenson

**Affiliations:** 1 Computing and Mathematical Sciences Department, Caltech, Pasadena, CA 91125, USA

## 

Many important processes in neurobiology as well as neuronal engineering applications rely upon multiresolution representation and analysis of external information. There are various approaches which attempt to explain how human perception systems perform multiscale representation and sparse coding. The model proposed here is based on a new approach to multiresolution of input signals and reveals synchronization as a general mechanism for multiscale representation common to various sensory systems. The proposed mechanism is nonlinear and adaptive in the sense that it does not rely on convolution with a preconceived basis. For the visual system this approach is a major departure from the current linear paradigm, which holds that the structure of the receptive fields and their variations are responsible for performing multiscale analysis. While there are some well-known, important roles played by entrainment in neuronal systems, our model reveals a new function of dynamic coordination in the brain - multiscale encoding, thus demonstrating that synchronization plays a greater role in perception in general and in vision in particular, than was previously thought.

